# BH3 Mimetics for the Treatment of Prostate Cancer

**DOI:** 10.3389/fphar.2017.00557

**Published:** 2017-08-18

**Authors:** Philipp Wolf

**Affiliations:** Department of Urology, Medical Center – University of Freiburg, Faculty of Medicine, University of Freiburg Freiburg, Germany

**Keywords:** prostate cancer, apoptosis, Bcl-2 proteins, BH3 mimetics, therapeutic resistance

## Abstract

Despite improved diagnostic and therapeutic intervention, advanced prostate cancer (PC) remains incurable. The acquired resistance of PC cells to current treatment protocols has been traced to apoptosis resistance based on the upregulation of anti-apoptotic proteins of the Bcl-2 family. The use of BH3 mimetics, mimicking pro-apoptotic activator or sensitizer proteins of the intrinsic apoptotic pathway, is therefore a promising treatment strategy. The present review gives an overview of preclinical and clinical studies with pan- and specific BH3 mimetics as sensitizers for cell death and gives an outlook how they could be effectively used for the therapy of advanced PC in future.

## Introduction

Prostate cancer remains the second most common cancer in men worldwide. About 1.1 million new cases are detected every year, accounting for 15% of all diagnosed cancers. As the fifth leading cause of cancer deaths, PC was responsible for an estimated 307,000 deaths representing 6.6% of the total cancer mortality (Ferlay et al., 2015). Locally restricted tumors can be successfully treated by radical prostatectomy, brachytherapy, external beam radiation, or active surveillance. In advanced PC, ADT is administered as first-line therapy. However, its median duration response of up to 18 months is limited, because virtually all patients develop CRPC with biochemical progress and high therapeutic resistance (Halabi et al., 2014; Katzenwadel and Wolf, 2015). In 2004, only a moderate overall survival benefit of about 3 months was reached in two independent phase 3 trials with docetaxel chemotherapy in metastatic CRPC (Petrylak et al., 2004; Tannock et al., 2004). Despite improved diagnostic and therapeutic procedures and improvements in terms of treatment sequencing and combinations, CRPC remains incurable. Studies evidence that a main factor for the acquired resistances to ADT, radiation, and chemotherapy is the resistance of PC cells to apoptosis (Gjertsen et al., 1998; Stein, 1999; DiPaola et al., 2001). Therefore, restoration of apoptosis represents a promising strategy for the future treatment of advanced PC.

## Regulation of Apoptosis by Bcl-2 Family Members

**FIGURE 1:**
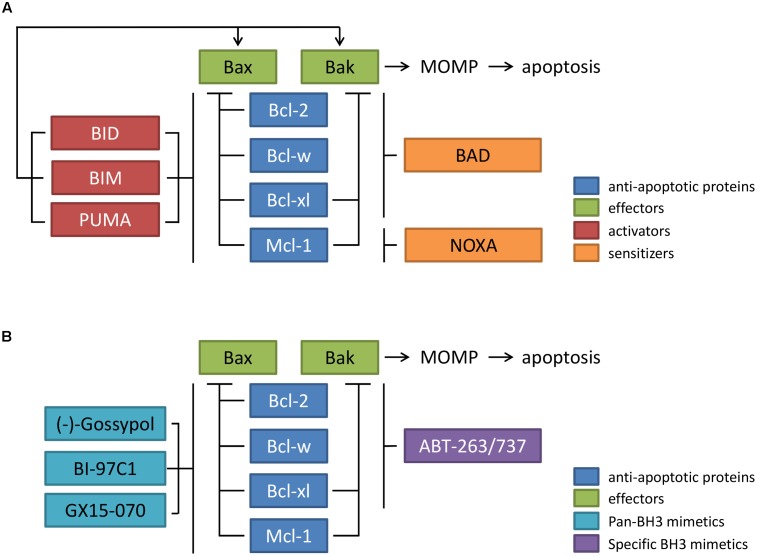
Schematic representation of the interactions between different members of the Bcl-2 protein family and BH3 mimetics. **(A)** The effector proteins of the Bcl-2 family, Bax and Bak, are inhibited by the anti-apoptotic proteins Bcl-2, Bcl-w, Bcl-xl, and Mcl-1. Upon induction of apoptosis, the sensitizers BAD and NOXA inhibit the anti-apoptotic proteins. The activators BID, BIM, and PUMA can also block the anti-apoptotic proteins and can interact directly with Bax and Bak. Free effectors can then induce MOMP and apoptosis. **(B)** The BH3 mimetics are surrogate proteins to the activator and sensitizer BH3-only proteins. They can be divided into pan-BH3 mimetics [GX15-070, (–)-gossypol, BI-97CI] antagonizing all members of the anti-apoptotic proteins, and specific BH3 mimetics (ABT-263/737), binding to Bcl-2, Bcl-xl, and Bcl-w, but not Mcl-1. Abbreviations: BAD, Bcl-2 antagonist of cell death; Bak, Bcl-2 antagonist/killer; Bax, Bcl-2 associated X protein; Bcl-2, B-cell lymphoma 2; Bcl-w, Bcl-2 like 2; Bcl-xl, B-cell lymphoma extra-large; BID, BH3 interacting domain death agonist; BIM, Bcl-2 interacting mediator of cell death; Mcl-1, myeloid cell leukemia sequence; MOMP, mitochondrial outer membrane permeabilization; NOXA, phorbol-12-myristate-13-acetate-induced protein 1; PUMA, p53 upregulated modulator of apoptosis. BH3 mimetics: ABT-263, Navitoclax; BI-97-CI, Sabutoclax; GX15-070, Obatoclax.

Apoptosis, the programmed cell death, is a highly regulated and controlled process of multicellular organisms for the elimination of surplus or damaged cells to preserve tissue and organ homeostasis (Kiraz et al., 2016). Key regulators of the intrinsic apoptotic pathway are proteins of the B-cell lymphoma 2 (Bcl-2) family (Youle and Strasser, 2008). According to their function in the apoptosis network, Bcl-2 family members can be divided into two different subgroups: the anti-apoptotic proteins (Bcl-2, Bcl-w, Bcl-xl, and Mcl-1) and the pro-apoptotic proteins. The latter group comprises the effectors (Bax and Bak), the activators (BID, BIM, and PUMA), and the sensitizers (BAD and NOXA) (**Figure 1A**). Structural analyses showed that the anti-apoptotic proteins share four regions of sequence homology dubbed Bcl-2 homology domains (BH1-4). The domains BH1-3 form a hydrophobic binding pocket into which the effectors can bind via their BH3 domain. The activator and sensitizer proteins hold only the BH3 domain and are therefore called BH3-only proteins (Czabotar et al., 2014).

In non-apoptotic cells, the effectors are inhibited by binding of the anti-apoptotic proteins. Upon induction of apoptosis, the BH3 only proteins are transcriptionally or post-translationally activated and bind via their BH3 domain to the binding pocket of the anti-apoptotic proteins to free the effectors. Taking different models into consideration, the activators are believed to directly activate Bax and Bak and to inhibit the anti-apoptotic proteins. The sensitizers, which cannot directly interact with the effectors, can also neutralize the anti-apoptotic proteins. Displacing Bax and Bak from the anti-apoptotic proteins leads to their homo-oligomerization and a formation of pores in the outer mitochondrial membrane. This event is termed mitochondrial outer membrane permeabilization (MOMP) and marks the point of no return in apoptosis. MOMP is followed by cytochrome c release from the mitochondria and activation of initiator caspase-9 and downstream effector caspases (Youle and Strasser, 2008).

## Apoptosis Resistance in Prostate Cancer

Resistance against apoptosis is a significant hallmark of cancer and contributes to tumor formation, survival, and therapeutic resistance (Cory et al., 2003; Hanahan and Weinberg, 2011). The acquired resistance of advanced PC to current treatment protocols (ADT, radiation, and chemotherapy) has been associated with apoptosis resistance of PC cells, especially based on an upregulation of the anti-apoptotic Bcl-2 family members (Gjertsen et al., 1998; Stein, 1999; Lebedeva et al., 2000; DiPaola et al., 2001). This has been proven by the fact that the treatment of hormone-naïve PC with ADT plus docetaxel, which primarily affects Bcl-2 expression by phosphorylation (Pienta, 2001; Boudny and Nakano, 2002), led to a survival benefit of about 13–15 months compared to the ADT monotherapy (Sweeney et al., 2015; Wolf, 2017).

In an immunohistological study, Bcl-2 was detected in 25% of human prostate adenocarcinomas and shown to be more present in high grade tumors (Gleason grade 8–10; 41%) and nodal metastases (38%) compared to lower grade primary tumors (Gleason 2–7; 16%; *P* < 0.05) (Krajewska et al., 1996). Bcl-2 expression was also associated with lower biochemical-free survival in patients with advanced PC undergoing ADT (Anvari et al., 2012).

Bcl-xl was detected in all tumors tested and more intense immunostaining was observed in the high grade primary tumors and in metastases compared to prostatic intraepithelial neoplasia (PIN) and low grade neoplasms (*P* < 0.0001). Moreover, it was more abundant in samples of patients with CRPC (Krajewska et al., 1996; Castilla et al., 2006).

Mcl-1 was expressed in 81% of the tumors, compared with only 38% cases of PIN (*P* < 0.001). A higher percentage of Mcl-1 positive cells was observed in high grade tumors and metastases than in lower grade tumors (*P* = 0.025) (Krajewska et al., 1996). Studies with different PC cell lines verified that, compared to Bcl-2, Bcl-xl and Mcl-1 protected the cells from different chemotherapeutic agents (Lebedeva et al., 2000; Reiner et al., 2015).

Interestingly, the pro-apoptotic effectors Bax and Bak were shown to be present in 95–100% and 77.5%, respectively, of all PCs tissues evaluated regardless of tumor grade (Krajewska et al., 1996; Yoshino et al., 2006; Anvari et al., 2012). Moreover, mutations of the Bak and Bax genes are rare events in PC (Yoshino et al., 2006).

## BH3 Mimetics for the Treatment of Prostate Cancer

### Pan-BH3 Mimetics

Due to their overexpression and their significant role in the induction of apoptosis, anti-apoptotic Bcl-2 proteins can act as suitable targets in cancer cells for the restoration of apoptosis. Bcl-2 family inhibition encompasses two main strategies: (i) knockdown and (ii) the use of synthetic low-molecular agents mimicking the BH3 only proteins. The latter are called BH3 mimetics or Bcl-2 inhibitors and can directly bind and thus inhibit the anti-apoptotic proteins (Scarfo and Ghia, 2013) (**Figure 1B**).

In preclinical and clinical studies against PC the natural BH3 mimetics (-)-Gossypol [R-(-)-enantiomer of gossypol, AT-101], BI-97C1 (Sabutoclax), and GX15-070 (Obatoclax) were used. They act as pan-Bcl-2 inhibitors targeting the four major anti-apoptotic Bcl-2 proteins Bcl-2, Bcl-xl, Mcl-1, and Bcl-w (Wolter et al., 2006; Lessene et al., 2008; Wei et al., 2010; Joudeh and Claxton, 2012) (**Table 1**). (-)-Gossypol alone inhibited cell growth and induced the intrinsic apoptosis of PC cells with 50% inhibitory concentration values (IC_50_) in the low μM range (Volate et al., 2010). Mechanistically, a blocking of the interactions of Bcl-xl with Bax or BAD and enhanced PUMA and NOXA levels were detected. Moreover, it synergistically increased the antitumor activity of docetaxel (Meng et al., 2008; Volate et al., 2010). The multikinase inhibitor Sorafenib synergistically suppressed the growth of PC cells in combination with (-)-gossypol by Mcl-1 inhibition and Bak activation (Lian et al., 2012). The use of valproic acid, a histone deacetylate inhibitor (HDACI), also heightened the cytotoxicity of (-)-gossypol. Mechanistically, valproic acid enhanced the induction of mitochondrial stress, as shown by upregulation of glycolysis- and hypoxia-associated proteins (Ouyang et al., 2011). (-)-Gossypol also acted as a radiosensitizer in a study of Xu et al. (2005). The pan-BH3 mimetic enhanced the radiation-induced apoptosis of PC-3 cells, which were established from a PC bone metastasis, show androgen-independent growth and express high levels of Bcl-2 and Bcl-xl (Kaighn et al., 1979). Combination therapy led to tumor regression in a PC-3 mouse xenograft model, and anti-CD31 immunostaining evidenced that the combination therapy also inhibited tumor angiogenesis (Xu et al., 2005). Synergistic effects by the pan-BH3 mimetic Obatoclax with androgen receptor (AR) inactivation by the antiandrogen bicalutamide was observed in a study of Santer et al. (2015). The combination of BI-97CI (Sabutoclax) with an IL-10 family cytokine, called melanoma differentiation associated gene-7/interleukin-24 (mda-7/Il-24), was marked by autophagy that facilitated NOXA and Bim-induced Bax/Bak mediated apoptosis. This resulted in an enhanced cytotoxicity in PC cells and significant *in vivo* inhibition of tumor growth (Dash et al., 2011).

**Table 1:** Preclinical studies of BH3 mimetics in combination with different agents against PC cells eliciting additive or synergistic cytotoxicity.

BH3 mimetic	Target	Combination with	Reference
**Pan-BH3 mimetics**			
(-)-Gossypol	Bcl-2, Bcl-xl, Bcl-w, Mcl-1	Docetaxel	Meng et al., 2008
(-)-Gossypol	Bcl-2, Bcl-xl, Bcl-w, Mcl-1	Sorafenib	Lian et al., 2012
(-)-Gossypol	Bcl-2, Bcl-xl, Bcl-w, Mcl-1	Valproic acid	Ouyang et al., 2011
(-)-Gossypol	Bcl-2, Bcl-xl, Bcl-w, Mcl-1	Radiation	Xu et al., 2005
GX15-070	Bcl-2, Bcl-xl, Bcl-w, Mcl-1	Bicalutamide	Santer et al., 2015
BI-97CI	Bcl-2, Bcl-xl, Bcl-w, Mcl-1	mda-7/IL-24	Dash et al., 2011
**Specific BH3 mimetics**			
ABT-263	Bcl-2, Bcl-xl, Bcl-w	Paclitaxel	Wang et al., 2015
ABT-737	Bcl-2, Bcl-xl, Bcl-w	Docetaxel	Parrondo et al., 2013
ABT-263/737	Bcl-2, Bcl-xl, Bcl-w	Docetaxel	Tamaki et al., 2014
ABT-737	Bcl-2, Bcl-xl, Bcl-w	Cisplatin	Bray et al., 2009
ABT-263	Bcl-2, Bcl-xl, Bcl-w	MLN2238	Wei et al., 2014
ABT-263	Bcl-2, Bcl-xl, Bcl-w	CP-d/n-ATF5-S1	Karpel-Massler et al., 2016
ABT-263/737	Bcl-2, Bcl-xl, Bcl-w	2DG	Yamaguchi et al., 2011
ABT-737	Bcl-2, Bcl-xl, Bcl-w	Pim kinase inhibitor	Song and Kraft, 2012
ABT-737	Bcl-2, Bcl-xl, Bcl-w	Pseudolaric acid B (PAB)	Tong et al., 2013
ABT-737	Bcl-2, Bcl-xl, Bcl-w	Transcriptional inhibitor ARC	Pandit and Gartel, 2010
ABT-737	Bcl-2, Bcl-xl, Bcl-w	MSeA	Yin et al., 2012
ABT-737	Bcl-2, Bcl-xl, Bcl-w	TRAIL	Song et al., 2008


AT-101 was also tested in clinical trials against PC; however, the goals of the studies were not reached. AT-101 as single agent had only limited activity in 23 patients with chemotherapy naïve CRPC. Two patients of a phase I/II study had a confirmed ≥ post-therapeutic prostate-specific antigen decline. No objective responses were observed. Gastrointestinal toxicity was the main adverse side effect and was dose-limiting (Liu et al., 2009). In a phase II trial, AT-101 was combined with docetaxel/prednisone treatment in 221 patients with hormone-naïve, progressive CRPC. The median overall survival of patients treated with AT-101 was 18.1 months and not significantly different from patients treated with docetaxel/prednisone alone (17.8 months; HR 1.07; 95% CI 0.72–1.55, *p* = 0.63) (Sonpavde et al., 2012).

It is discussed that the low antitumor activity of the pan-BH3 mimetics and their dose-limiting adverse side effects in clinical trials might be based on their moderate affinity and low specificity for the anti-apoptotic Bcl-2 proteins (Vogler et al., 2009). For example, gossypol compounds were shown to induce apoptosis in BAX/BAK-deficient cells and elicited several off-target effects. It is therefore suggested that they might have more functions than only to act as pan-BH3 mimetics (Billard, 2013). The main focus is therefore actually on the use of specific BH3 mimetics.

### Specific BH3 Mimetics

Specific BH3 mimetics, which were tested in PC cells to date, are ABT-737 and its orally administrable analog ABT-263 (Navitoclax). They are BAD-like BH3 mimetics, which were rationally designed by structure-based drug design for binding the hydrophobic groove of Bcl-xl (Oltersdorf et al., 2005). Like BAD, ABT-263/737 selectively bind with subnanomolar affinities to Bcl-2, Bcl-xl, and Bcl-w, but not to Mcl-1 (**Figure 1B**) (Zhai et al., 2006). This means that ABT-263/737 are highly specific and cancer cells with high overexpression of endogenous Bcl-2 or Bcl-xl are particularly vulnerable.

An overview of preclinical studies with ABT-263/737 against PC is given in **Table 1**. Since the survival of prostate tumor cells is mainly dependent on Bcl-xl and Mcl-1, the rationale was given to combine the BH3 mimetics with agents that additionally degrade or neutralize Mcl-1 for the induction of apoptosis.

Some authors used the combination of ABT-263/ABT-737 with different chemotherapeutic agents to enhance efficacy and to overcome resistance. Wang and colleagues proved an enhanced Bcl-xl level as the reason for paclitaxel resistance in PC cells (Wang et al., 2015). Combination with ABT-263, which inhibited Bcl-xl, triggered the paclitaxel induced apoptosis. Different sensitivities to the BH3 mimetic were detected in LNCaP and PC-3 cells, although both lines showed similar expression of the Bcl-2 family proteins. The authors therefore speculated that downstream elements, like unidentified proteins affecting MOMP, could be responsible for this observation (Wang et al., 2015). Docetaxel was found to increase cyclin B1/Cdk1-mediated phosphorylation of Bcl-2 and Bcl-xl and to decrease Mcl-1. This, however, was not enough alone to counteract the high levels of the anti-apoptotic proteins in PC-3 cells. Therefore, docetaxel was combined with ABT-263/737, which led to an enhanced induction of apoptosis (Parrondo et al., 2013; Tamaki et al., 2014). Bray and colleagues developed a PC mouse model with tumorigenesis and apoptosis resistance based on overexpression of Bcl-2. They could show that taxane-mediated Bim induction was insufficient to exceed the apoptotic threshold conferred by Bcl-2 and used ABT-737 for chemosensitization of the tumors (Bray et al., 2009).

ABT-263 was also combined with the proteasome inhibitor MLN2238. Proteasome inhibitors are able to modulate pro-apoptotic factors, like p53 and NOXA, for the induction of apoptosis. Whereas ABT-263 and MLN2238 alone only showed a mild cytotoxicity in the highly metastatic and androgen-independent PC cell lines PC-3 and C4-2B, their combination led to synergistic effects. Molecular examinations showed that MLN2238 enhanced the NOXA levels, leading to an enhanced NOXA/Mcl-1 formation and dissociation of Bax from Mcl-1 (Wei et al., 2014).

A synthetic cell penetrating peptide, called CP-d/n-AFT5-S1, was used to inhibit the transcription factor 5 (ATF5) (Karpel-Massler et al., 2016). ATF5 belongs to the cAMP response-element binding protein (CREB) family and regulates the transcription of Bcl-2 and Mcl-1 (Chen et al., 2012; Izumi et al., 2012). It is upregulated in many cancers and promotes apoptosis resistance (Monaco et al., 2007). CP-d/n-AFT5-S1 led to diminished levels of Bcl-2 and Mcl-1 in cancer cells of different origin, including PC, and acted synergistically with ABT-263. In a mouse model the combination therapy significantly reduced the growth of PC-3 tumor xenografts. Mechanistically, a decreased expression of the Mcl-1 interacting proteins Bag3 and Usp9X was observed after incubation with the inhibitor, which was followed by Mcl-1 depletion. Interestingly, CP-d/n-AFT5-S1 also increased the Bcl-xl expression in some cell lines. The authors therefore discussed the possibility that the Bcl-xl overexpression could render cancer cells more sensitive to ABT-263 (Karpel-Massler et al., 2016).

In a further study, 2-deoxyglucose (2DG) was successfully combined with ABT-263/737 and inhibited the growth of mouse prostate tumor xenografts. It was found that 2DG primed the cells by interference with Mcl-1/Bak complexes, making it easier for the BH3 mimetics to free Bak from Bcl-2 (Yamaguchi et al., 2011).

Pim serine/threonine kinases promote the tumorigenesis of PC cells and contribute to the therapeutic resistance. Pim kinase inhibitors acted synergistically with ABT-737 *in vitro* and *in vivo*. The study proved that the Pim kinase inhibitors decreased the Mcl-1 levels by blocking 5′-cap dependent translation and reduction of the protein half-life. Moreover, they led to an enhanced transcription of NOXA, which blocked the remaining levels of Mcl-1 (Song and Kraft, 2012).

Pseudolaric acid B (PAB) is a plant-derived terpenoid, which shows antitumorous or chemopreventive activity in many types of cancer. PAB induced cell cycle arrest as well as a downregulation of Bcl-2 and Mcl-1 via the JNK-mediated cell death pathway, which is known to activate pro-apoptotic and to inactivate anti-apoptotic Bcl-2 family proteins. As a result PAB acted synergistically with ABT-737 in PC cells (Tong et al., 2013).

The transcriptional inhibitor ARC (4-amino-6-hydrazino-7-β-D-ribofuranosyl-7H-pyrrolo(2,3-d)-pyrimidine-5-carboxamide) is an inhibitor of the P-TEFb kinase (CDK9/CyclinT1 complex). It was demonstrated that ARC effectively downregulated the Mcl-1 expression of PC cells and showed synergistic effects with ABT-737 (Pandit and Gartel, 2010).

The selenium compound methylseleninic acid (MSeA) has been reported to downregulate prostate-specific antigen expression via disruption of AR signaling (Dong et al., 2004; Zhao et al., 2004). MSeA led to a decreased Mcl-1 expression in DU145 cells and acted therefore synergistically with ABT-737. Remarkably, synergism was due to a dephosphorylation of BAD by MSeA, since phosphorylation of BAD at ser-136 and ser-112 was identified as an ABT-737 resistance mechanism (Yin et al., 2012).

Song and colleagues demonstrated that ABT-737 can directly influence the extrinsic apoptotic pathway (Song et al., 2008). Combination of the tumor necrosis factor-related apoptosis-inducing ligand (TRAIL) with ABT-737 led to a synergistic cytotoxicity in PC cells. Interestingly, ABT-737 treatment was shown to enhance the expression of the TRAIL receptor DR5 via a transcriptional mechanism dependent on the NF-kappaB site of the DR5 promotor (Song et al., 2008).

Testing of ABT-737 in clinical trials against PC is ongoing. Recently, a phase II study of ABT-263 in combination with abiraterone or abiraterone/hydroxychloroquine in patients with CRPC following chemotherapy and abiraterone treatment has been terminated (Identifier: NCT01828476)^1^. Study results have not been published yet.

## Conclusions and Outlooks

BH3 mimetics seem to be particularly suitable for the combination treatment of PC for several reasons. First, different preclinical studies have proven that BH3 mimetics are sensitizers for apoptosis in PC cells, which represent advanced stages of the disease and which are known to be resistant against apoptosis. Second, the intrinsic apoptotic pathway is involved in cell death caused by most of the chemotherapeutic drugs, toxins, antiandrogens, and irradiation, and BH3 mimetics are able to lower the threshold for its activation. Third, BH3 mimetics directly abrogate the interaction between pro- and anti-apoptotic Bcl-2 proteins, with a direct activation of the effectors Bax and Bak. This might be more promising than the use of antitumor drugs that act upstream. Fourth, Bax and Bak are expressed in nearly all PC cells in a non-mutated form and can therefore successfully be activated for the induction of apoptosis.

It is important to ensure the comprehensive blocking of all of the anti-apoptotic members of the Bcl-2 family for highest efficacy. This is in principle possible by using pan Bcl-2 inhibitors. However, these molecules have a lower affinity to the target proteins than the specific Bcl-2 inhibitors, and high off-target side effects led to a lack of efficacy and to dose-limiting toxicities in clinical trials.

Specific BH3 mimetics like ABT-737 have the advantage to bind with higher affinity; however, might not be sufficient to induce apoptosis. Moreover, upregulation of non-targeted Bcl-2 members could lead to resistance (Konopleva et al., 2006; Yecies et al., 2010). Specific Mcl-1 inhibitors (Small molecule Mcl-1 inhibitor, MIM-1, TW-37, A1210477, biphenyl-NOXA BH3 peptide) are therefore currently under investigation to be combined (Besbes et al., 2015).

Since BH3 mimetics developed so far are non-targeted molecules, off-target adverse side effects could endanger the success of clinical trials. The best way should therefore be to combine BH3 mimetics with tumor-specific agents to selectively attack the cancer cells. One such agent can be an immunotoxin consisting of an antibody fragment specifically binding to a tumor surface antigen and of the cytotoxic domain of *Pseudomonas aeruginosa* exotoxin A (PEA). PEA-based immunotoxins inhibit the protein biosynthesis of antigen-expressing cancer cells and especially downregulate Mcl-1 (Michalska and Wolf, 2015). Such immunotoxins, targeting mesothelin on pancreatic cancer cells or the transferrin receptor on small cell lung cancer cells, were successfully combined with ABT-263/737 and induced death in cells that were shown to be resistant against the individual components (Mattoo and FitzGerald, 2013; Hollevoet et al., 2014). **Table 1** demonstrates that only unspecific agents were added to the BH3 mimetics in the studies against PC. The combination of specific BH3 mimetics with targeted molecules, leading to an induction of apoptosis specifically in cancer cells, could therefore provide a successful route for an improved therapy of PC in future.

## Author Contributions

PW prepared the whole manuscript.

## Conflict of Interest Statement

The author declares that the research was conducted in the absence of any commercial or financial relationships that could be construed as a potential conflict of interest.

## Footnotes

1 ClinicalTrials.gov

## Abbreviations

2DG: 2-deoxyglucose
ADT: androgen deprivation therapy
CRPC: castration resistant prostate cancer
JNK: c-Jun N-terminal kinase
MSeA: methylseleninic acid
PAB: pseudolaric acid
PC: prostate cancer
